# CCAT1 promotes triple-negative breast cancer progression by suppressing miR-218/ZFX signaling

**DOI:** 10.18632/aging.102080

**Published:** 2019-07-16

**Authors:** Chunyong Han, Xuebiao Li, Qian Fan, Guangshu Liu, Jian Yin

**Affiliations:** 1 Department of Breast Reconstruction, The Sino-Russian Joint Research Center for Oncoplastic Breast Surgery, Tianjin Medical University Cancer Institute and Hospital, National Clinical Research Center for Cancer, Key Laboratory of Cancer Prevention and Treatment of Tianjin, Tianjin Clinical Research Center for Cancer, Key Laboratory of Breast Cancer Prevention and Therapy, Ministry of Education, Tianjin 300060, China; 2 Department of Cardiovascular Surgery, Second Affiliated Hospital, College of Medicine, Zhejiang University, Hangzhou 310009, China; 3 Department of Lymphoma, Tianjin Medical University Cancer Institute and Hospital, National Clinical Research Center of Cancer, Key Laboratory of Cancer Prevention and Treatment of Tianjin, Tianjin 300060, China; 4 Department of Oncology, People's Hospital of Langfang City, Langfang 65000, China

**Keywords:** lncRNA CCAT1, miR-218, ZFX, triple-negative breast cancer (TNBC)

## Abstract

Long non-coding RNAs (lncRNAs) regulate cancer development and progression. Here, we investigated the role of the lncRNA CCAT1 in triple-negative breast cancer (TNBC). CCAT1 expression was higher in TNBC cells than normal breast epithelial cells. Additionally, CCAT1 expression was higher in TNBC patient tumor tissue than adjacent normal breast tissue. Silencing CCAT1 inhibited TNBC cell proliferation, migration, and invasion *in vitro*, and tumor growth and progression *in vivo*. Bioinformatics analysis revealed that microRNA-218 (miR-218) is a potential target of CCAT1. Silencing CCAT1 resulted in an increase in miR-218 expression and inhibited TNBC cell proliferation, migration, and invasion. Silencing miR-218 reversed the effects of CCAT1 knockdown on cell proliferation, migration, and invasion, suggesting that CCAT1 promotes TNBC progression by downregulating miR-218 expression. We identified the zinc finger protein ZFX as a putative downstream target of miR-218 through bioinformatics analysis. ZFX expression was higher in TNBC than normal breast cell lines and higher in TNBC tumor tissue than adjacent normal breast tissue. Overexpression of ZFX reversed the tumor-suppressive effects of miR-218 on TNBC cell proliferation, migration, and invasion. Our data indicate that CCAT1 promotes TNBC progression by targeting the miR-218/ZFX axis.

## INTRODUCTION

Breast cancer is the most common cancer among women and the leading cause of cancer-related death worldwide [1]. The incidence and mortality in breast cancer has increased over the past few decades in China [2]. Although breast cancer is a heterogeneous disease, it can be divided into four major molecular subtypes: luminal A, luminal B, triple-negative/basal-like, and HER2-enriched [3]. Triple-negative breast cancer (TNBC) is characterized by the absence of estrogen and progesterone receptors, and a lack of HER2 overexpression. TNBC is the most aggressive of the four molecular subtypes and has been associated with a higher incidence of local recurrence and metastasis. There are few targeted therapies for TNBC. Treatment generally involves a combination of surgery, chemotherapy, radiotherapy, and/or immunotherapy [4, 5]. However, the prognosis of patients with TNBC (particularly those with advanced-stage disease) is poor [4]. Therefore, it is important to understand the mechanisms underlying TNBC progression in order to develop more effective therapeutic strategies.

Long non-coding RNAs (lncRNAs) are RNA transcripts with lengths of at least 200 nucleotides that do not encode proteins [6, 7]. They have crucial roles in regulating gene expression in various biological processes, including organ development, cell fate, and carcinogenesis. Additionally, they can regulate cell proliferation, differentiation, and apoptosis [8]. Aberrant expression of lncRNAs has been associated with cancer development and progression [9]. For example, downregulation of the lncRNA RP11-766N7.4 correlates with esophageal squamous cell carcinoma initiation and metastasis [10], while upregulation of the lncRNA DANCR promotes gastric cancer initiation and progression [11]. Finally, the lncRNA HULC promotes liver cancer progression by regulating microRNA (miR)-186/HMGA2 signaling [12], while the lncRNA FOXD2-AS1 promotes colorectal cancer cell proliferation through an interaction with miR-185-5p [13].

LncRNAs are frequently dysregulated in breast cancer. For example, upregulation of the lncRNA ROR promotes tamoxifen resistance by inducing autophagy [14]. Additionally, upregulation of SNHG15 expression promotes cell proliferation, migration, and invasion by sponging miR-211-3p [15]. Downregulation of the lncRNA XIST inhibits breast cancer cell growth, migration, and invasion via the miR-155/CDX1 axis [16]. Dysregulation of several other lncRNAs, including ANRIL [17], MALAT1 [18], snaR [19], and RoR [20], has also been observed in TNBC.

Colon cancer-associated transcript 1 (CCAT1, 2,628 base pairs in length) is a lncRNA that has been associated with colorectal, gastric, hepatic, and ovarian carcinogenesis [21]. Overexpression of CCAT1 has been observed in colorectal cancer, where it promotes tumor growth and metastasis [22, 23]. Similarly, CCAT1 is overexpressed in gastric cancer tissue and is associated with tumor progression [24, 25]. One study has demonstrated an association between CCAT1 and both overall and progression-free survival in breast cancer [26]. It is also associated with resistance to radiotherapy [27]. In the present study, we investigated the role of CCAT1 in TNBC progression.

## RESULTS

### CCAT1 is upregulated in human TNBC cells and tissue

**Figure 1:**
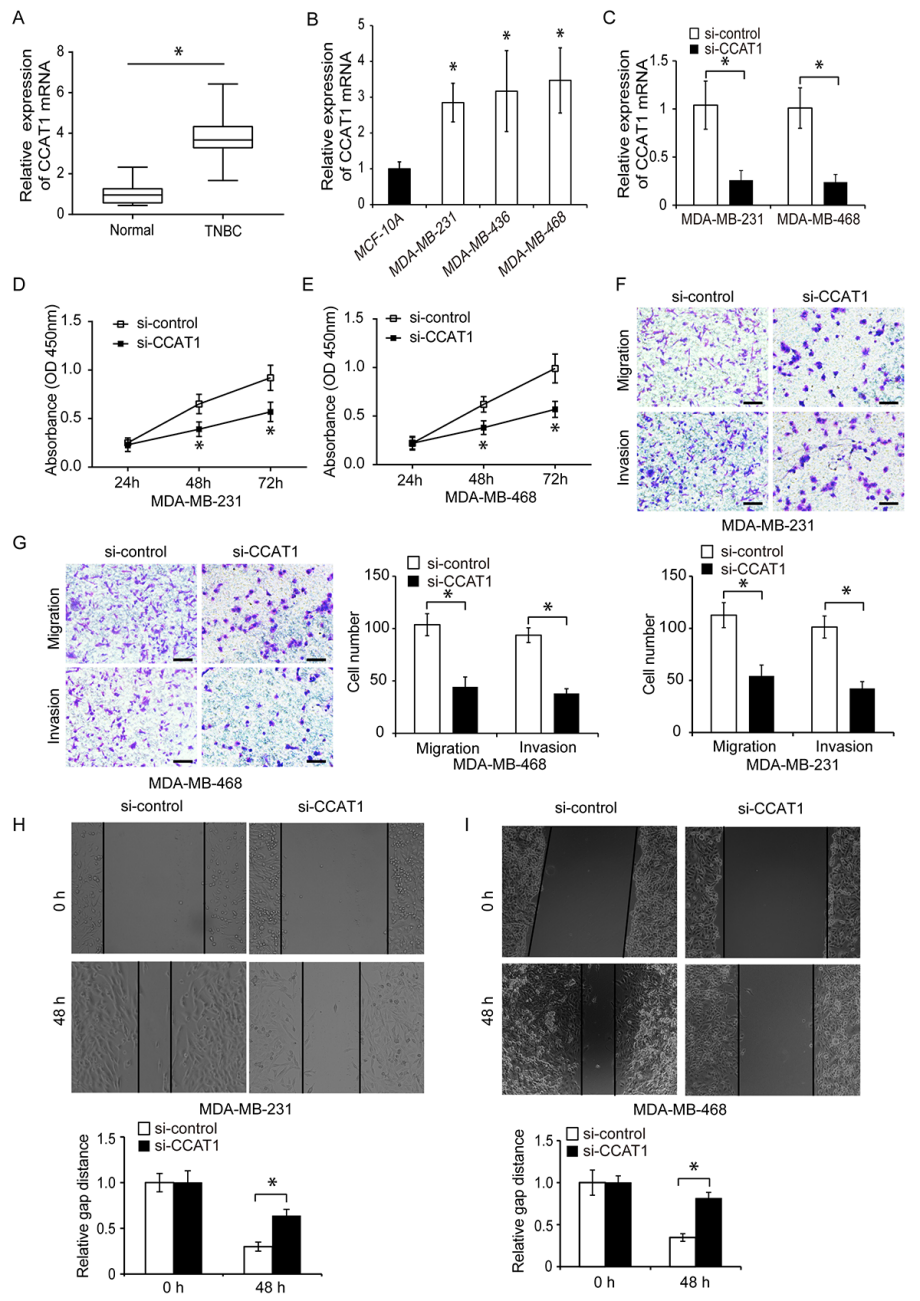
**CCAT1 is upregulated in human TNBC cells and promotes proliferation, migration, and invasion *in vitro*.** (**A**) Quantitative RT-PCR analysis of relative CCAT1 expression in human TNBC and adjacent normal tissue. (**B**) Quantitative RT-PCR analysis of relative CCAT1 expression in three TNBC cell lines (MDA-MB-231, MDA-MB-436, and MDA-MB-468) and in a normal human breast epithelial cell line (MCF-10A). (**C**) Quantitative RT-PCR analysis of relative CCAT1 expression in TNBC cells transfected with either si-CCAT1 or si-control. (**D**, **E**) CCK8 assays of cell proliferation following transfection of TNBC cells with either si-CCAT1 or si-control. (**F**, **G**) Analysis of the migration and invasion capacities of MDA-MB-231 and MDA-MB-468 cells following transfection with either si-CCAT1 or si-control. (**H**, **I**) Analysis of the migration capacity of MDA-MB-231 and MDA-MB-468 cells following transfection with either si-CCAT1 or si-control using wound healing assays. Scale bars, 200 μm. *P < 0.05 compared to controls.

We evaluated CCAT1 expression in human TNBC and adjacent normal tissue using quantitative real-Time PCR **(**qRT-PCR). CCAT1 expression was higher in human TNBC tissue compared to adjacent normal tissue (Figure 1A, P < 0.05). We then analyzed CCAT1 expression in three different TNBC cell lines (MDA-MB-231, MDA-MB-436, and MDA-MB-468) and one normal breast epithelial cell line (MCF-10A). CCAT1 was upregulated in all TNBC cell lines compared to controls (Figure 1B, P < 0.05).

### CCAT1 promotes TNBC cell proliferation, migration, and invasion

**Figure 2:**
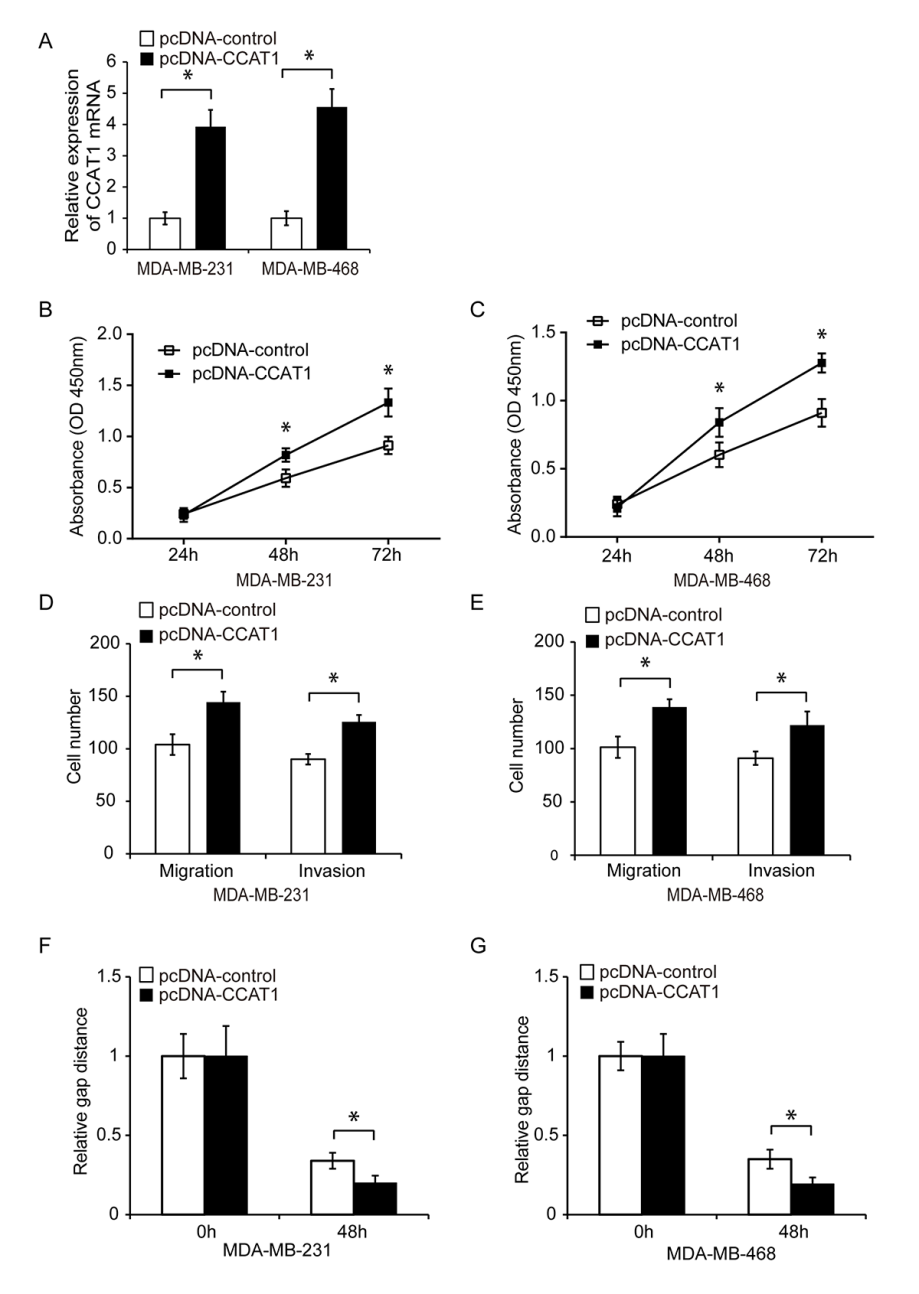
**Overexpression of CCAT1 promotes TNBC cell proliferation, migration, and invasion.** (**A**) Quantitative RT-PCR analysis of relative CCAT1 expression in TNBC cells transfected with either pcDNA-CCAT1 or pcDNA-control. (**B**, **C**) Analysis of TNBC cell proliferation following transfection with either pcDNA-CCAT1 or pcDNA-control using CCK8 assays. (**D**, **E**) Analysis of the migration and invasion capacities of MDA-MB-231 and MDA-MB-468 cells transfected with pcDNA-CCAT1 or pcDNA-control using transwell assays. (**F**, **G**) Analysis of the migration capacity of MDA-MB-231 and MDA-MB-468 cells transfected with pcDNA-CCAT1 or pcDNA-control using wound healing assays. *P < 0.05 compared to controls.

We next investigated whether CCAT1 could contribute to TNBC progression. CCAT1 was knocked down in MDA-MB-231 and MDA-MB-468 cells using siRNA. Downregulation of CCAT1 expression in both cell lines was confirmed using qRT-PCR (Figure 1C, P < 0.05). The effects of CCAT1 on cell proliferation were analyzed 24, 48, and 72 hours after siRNA transfection using CCK8 assays. CCAT1 knockdown resulted in reduced proliferation of TNBC cells compared to controls (Figure 1D and 1E, P < 0.05). We then analyzed whether CCAT1 was important for TNBC cell migration and invasion. Transwell and wound healing assays demonstrated that CCAT1 knockdown reduced cell invasion and migration in TNBC cells compared to controls (Figure 1F–1I, P < 0.05). We transfected TNBC cells with either a plasmid expressing CCAT1 (pcDNA-CCAT1) or empty vector control (pcDNA-control) and analyzed the effects of CCAT1 overexpression on cell proliferation using CCK8 assays. The transfection efficiency was confirmed by qRT-PCR (Figure 2A, P < 0.05). Overexpression of CCAT1 enhanced TNBC proliferation (Figure 2B and 2C, P < 0.05). Additionally, transwell and wound healing assays demonstrated that overexpression of CCAT1 promoted TNBC cell invasion and migration (Figure 2D–2G, P < 0.05). These findings indicated that CCAT1 promoted key steps in TNBC progression *in vitro*.

### CCAT1 acts as a sponge for miR-218

**Figure 3:**
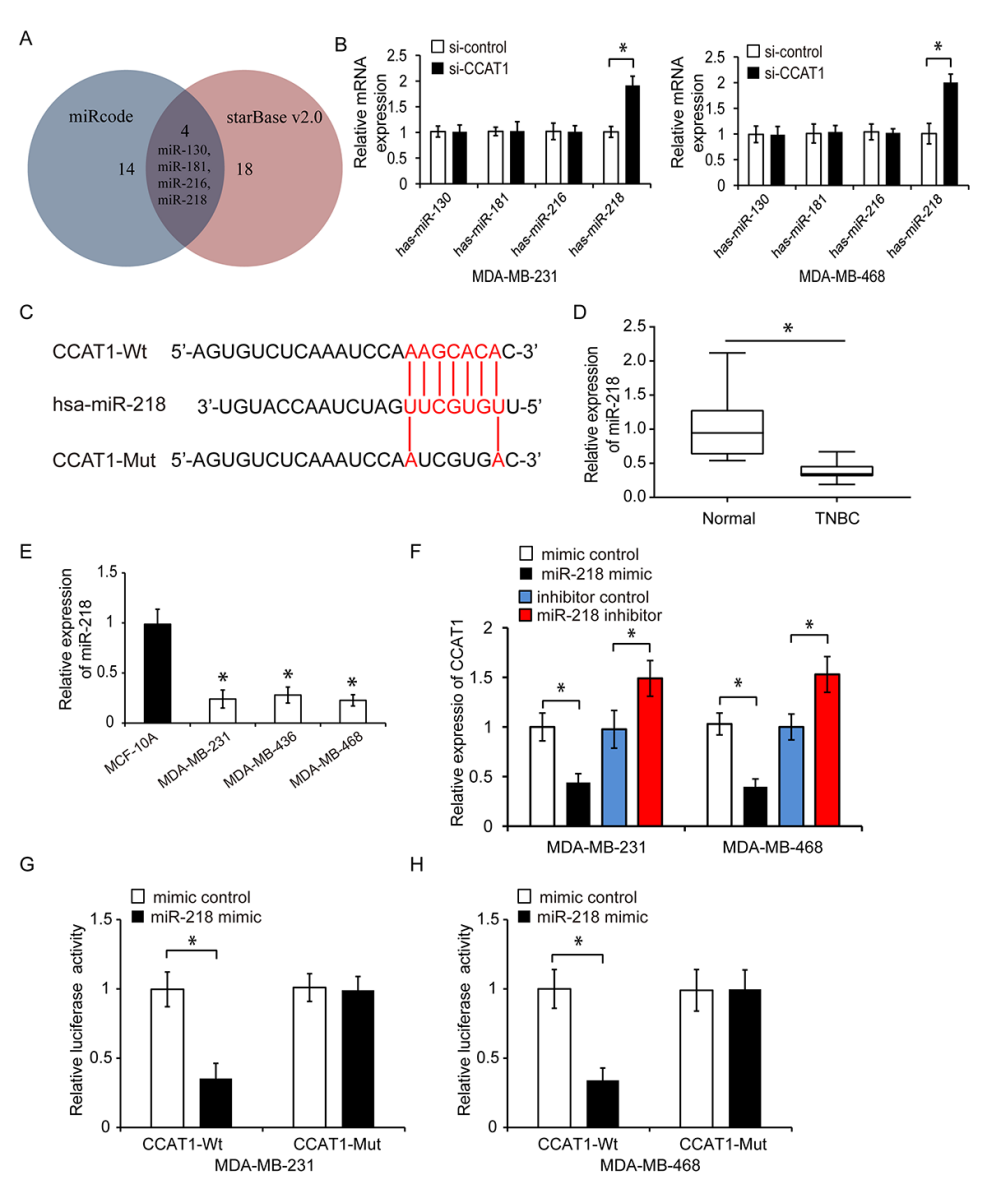
**CCAT1 functions as a sponge for miR-218.** (**A**) MiRcode and starBase were used to predict the miRNAs that could bind to CCAT1. Four miRNAs were identified: miR-130, miR-181, miR-216 and miR-218. (**B**) Relative expression of these four miRNAs in TNBC cells following transfection with si-CCAT1 or si-control. (**C**) Diagram showing the predicted miR-218 binding site in the CCAT1 sequence and the nucleotides that were mutated to impair binding. (**D**) Analysis of miR-218 expression in human TNBC and adjacent normal tissue by qRT-PCR. (**E**) Analysis of the relative expression of miR-218 in three TNBC cell lines (MDA-MB-231, MDA-MB-436, and MDA-MB-468) and in a human normal breast epithelial cell line (MCF-10A) by qRT-PCR. (**F**) Relative expression of CCAT1 after transfection of TNBC cells with either a miRNA mimic control, miR-218 mimic, inhibitor control, or miR-218 inhibitor. (**G**, **H**) Luciferase reporter assays demonstrating that overexpression of miR-218 repressed the luciferase activity in MDA-MB-231 and MDA-MB-468 cells transfected with CCAT1-Wt. *P < 0.05 compared to controls.

We next investigated the mechanisms underlying the effects of CCAT1 on TNBC cell proliferation, migration, and invasion. We performed bioinformatics analysis using miRcode and starBase 2.0 to identify potential miRNA targets of CCAT1 (Figure 3A). This analysis revealed four miRNAs (miR-130, miR-181, miR-216, and miR-218) that were potential targets of CCAT1. We transfected MDA-MB-231 and MDA-MB-468 cells with CCAT1 siRNA and found that only miR-218 expression was increased in response to CCAT1 knockdown (Figure 3B, P < 0.05). Therefore, we hypothesized that CCAT1 could function by targeting miR-218 in TNBC (Figure 3C). We evaluated miR-218 expression in human TNBC cell lines using qRT-PCR. MiR-218 was downregulated in all TNBC cell lines and tumor tissue analyzed (Figure 3D and 3E, P < 0.05). Overexpression of miR-218 resulted in a reduction in CCAT1 expression while knockdown of miR-218 resulted in an increase in CCAT1 expression in MDA-MB-231 and MDA-MB-468 cells (Figure 3F, P < 0.05). We constructed a luciferase reporter containing either wild-type CCAT1 (CCAT1-Wt) or a mutant version of CCAT1 (CCAT1-Mut) in which the binding sites for miR-218 were mutated. Overexpression of miR-218 inhibited luciferase activity in MDA-MB-231 and MDA-MB-468 cells transfected with CCAT1-Wt but not in cells transfected with CCAT1-Mut (Figure 3G and 3H, P < 0.05). These data suggested that CCAT1 could act as sponge for miR-218.

### Overexpression of miR-218 inhibits TNBC cell proliferation, migration, and invasion

**Figure 4:**
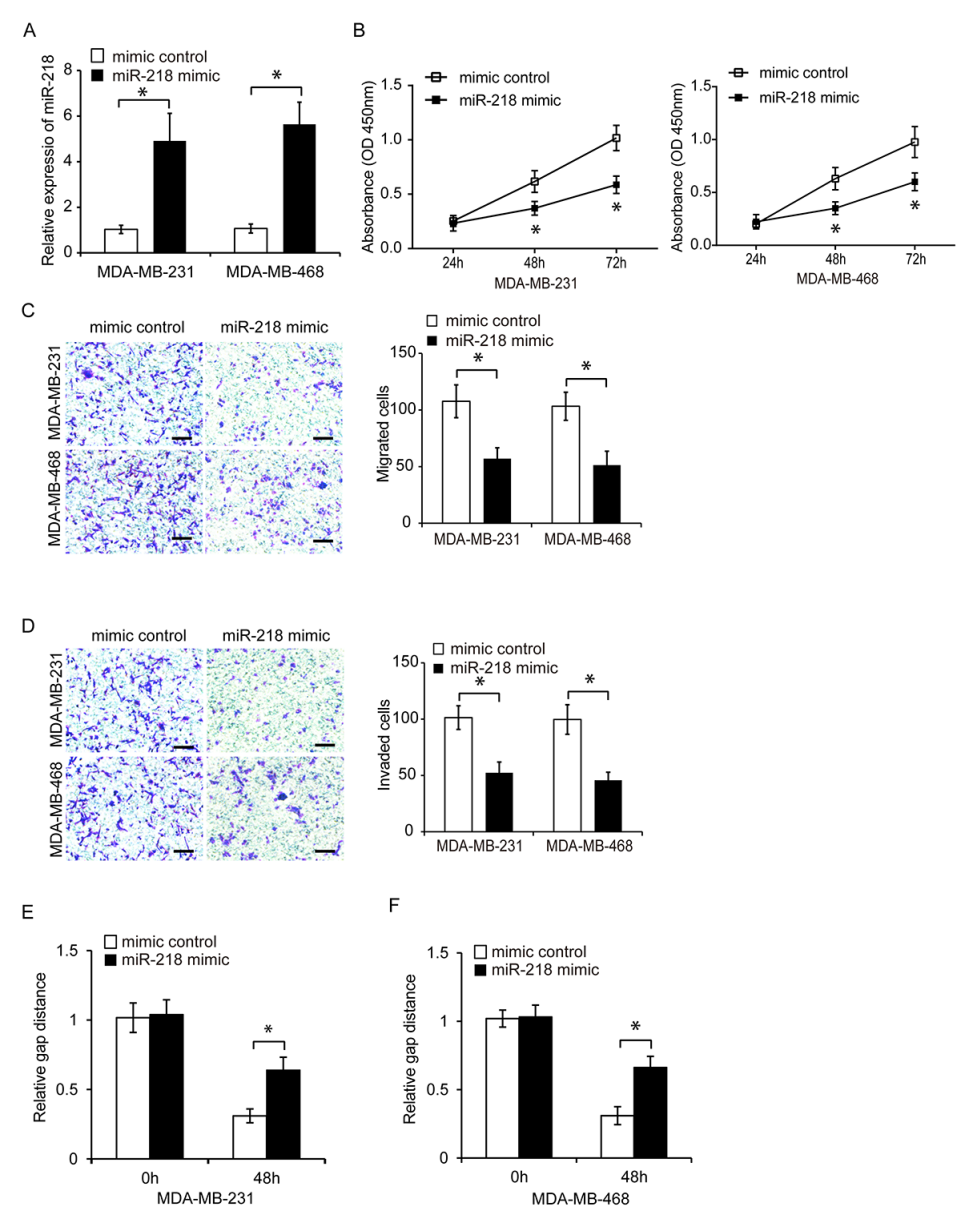
**Overexpression of miR-218 suppresses TNBC cell proliferation, migration, and invasion.** (**A**) Relative miR-218 expression in TNBC cells following transfection with a miRNA mimic or miR-218 mimic control. (**B**) Analysis of TNBC cell proliferation following transfection with a miR-218 mimic or miRNA mimic control using CCK8 assays. (**C**, **D**) Analysis of the migration and invasion capacities of MDA-MB-231 and MDA-MB-468 cells following transfection with a miR-218 mimic or miRNA mimic control using transwell assays. (**E**, **F**) Analysis of the migration capacity of MDA-MB-231 and MDA-MB-468 cells following transfection with either a miR-218 mimic or miRNA mimic control using wound healing assays. Scale bars, 200 μm. *P < 0.05 compared to controls.

We next investigated the effects of miR-218 on TNBC cell proliferation. MiR-218 was overexpressed in MDA-MB-231 and MDA-MB-468 cells. Treatment of TNBC cells with a miR-218 mimic resulted in increased expression of endogenous miR-218 (Figure 4A, P < 0.05). CCK8 assays of cell proliferation revealed that overexpression of miR-218 reduced TNBC cell proliferation (Figure 4B, P < 0.05). We also evaluated the effects of miR-218 on cell invasion and migration using transwell and wound healing assays, respectively. Overexpression of miR-218 resulted in a decrease in TNBC cell invasion and migration compared to controls (Figure 4C–4F, P < 0.05). These data suggested that miR-218 has a tumor-suppressive role in TNBC.

### CCAT1 promotes TNBC cell proliferation, migration, and invasion by inhibiting miR-218

**Figure 5:**
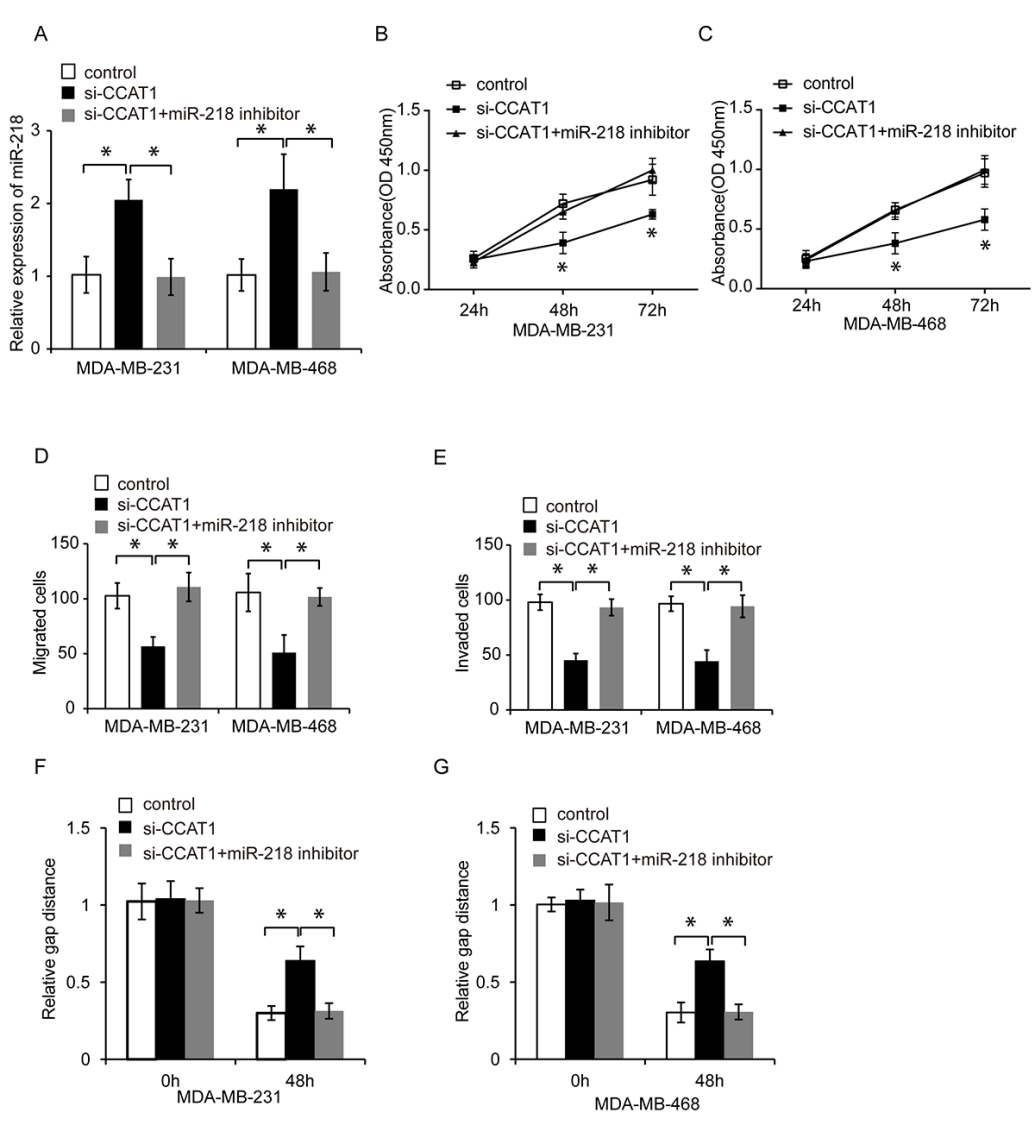
**CCAT1 promotes TNBC cell proliferation, migration, and invasion by inhibiting miR-218.** (**A**) Quantitative RT-PCR analysis of relative miR-218 expression in TNBC cells following transfection with control, si-CCAT1, or si-CCAT1 + a miR-218 inhibitor. (**B**, **C**) CCK8 assays of MDA-MB-231 and MDA-MB-468 cell proliferation following transfection with either control, si-CCAT1, and si-CCAT1 + a miR-218 inhibitor. (**D**, **E**) Analysis of the migration and invasion capacities of MDA-MB-231 and MDA-MB-468 cells transfected with control, si-CCAT1, or si-CCAT1 + a miR-218 inhibitor using transwell assays. (**F**, **G**) Analysis of the migration capacity of MDA-MB-231 and MDA-MB-468 cells transfected with control, si-CCAT1, or si-CCAT1 + a miR-218 inhibitor using wound healing assays. *P < 0.05 compared to controls.

We next investigated whether CCAT1 promoted TNBC progression by inhibiting miR-218. We knocked down miR-218 expression in CCAT1-silenced TNBC cells and analyzed the effects on cell proliferation, migration, and invasion using CCK8, wound healing, and transwell assays, respectively (Figure 5A, P < 0.05). Inhibition of miR-218 abrogated the suppressive effects of CCAT1 knockdown on TNBC cell proliferation, migration, and invasion (Figure 5B–5G, P < 0.05). These results indicated that CCAT1 promoted TNBC cell proliferation, migration and invasion via inhibiting miR-218.

### MiR-218 directly targets ZFX in TNBC cells

**Figure 6:**
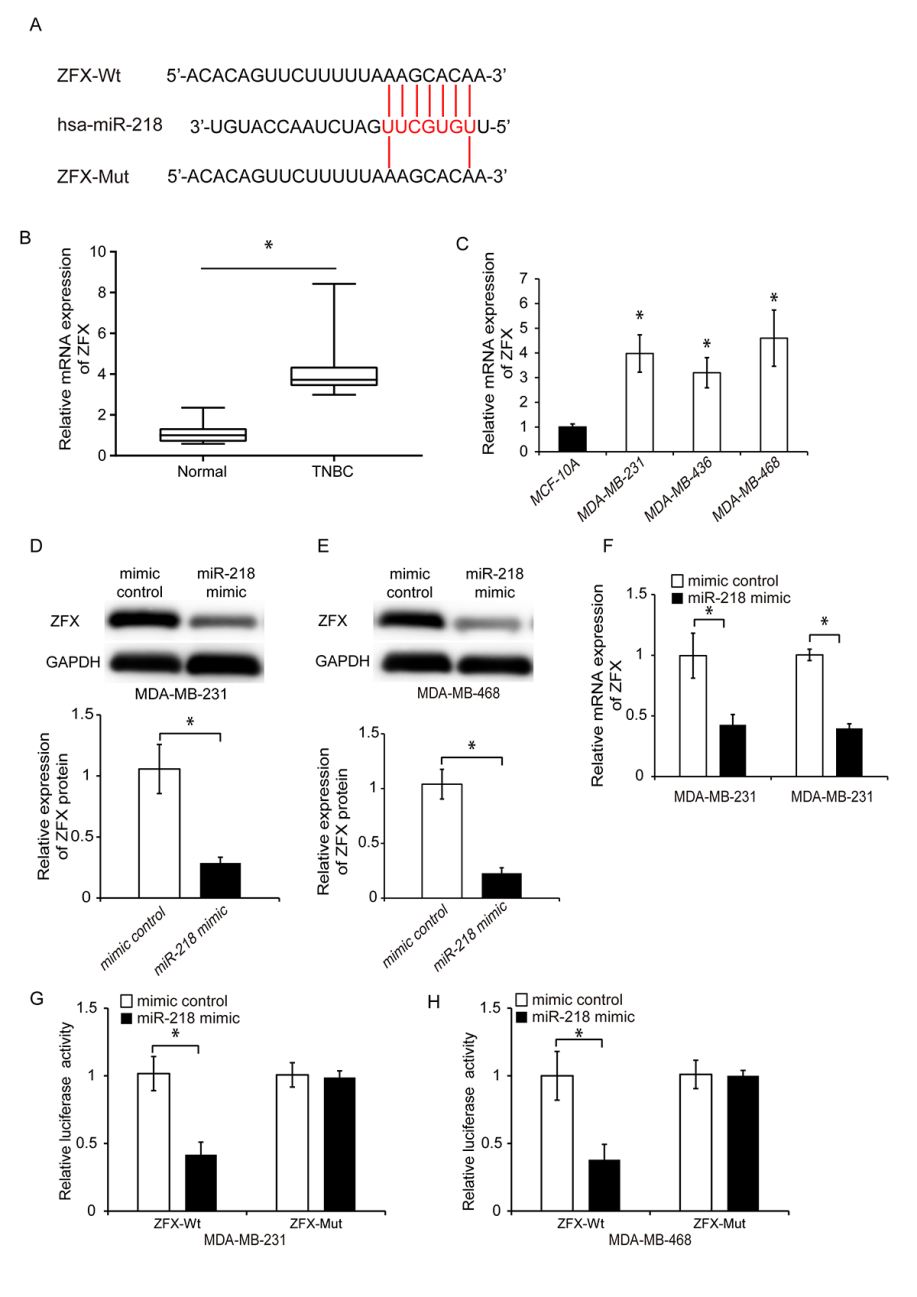
**MiR-218 directly targets ZFX in TNBC cells.** (**A**) Diagram showing the predicted miR-218 binding site in the ZFX sequence and the nucleotides that were mutated to impair binding. (**B**) Analysis of relative ZFX mRNA expression in human TNBC and adjacent control tissue by qRT-PCR. (**C**) Quantitative RT-PCR analysis of the relative expression of ZFX in three TNBC cell lines (MDA-MB-231, MDA-MB-436, and MDA-MB-468) and in a normal human breast epithelial cell line (MCF-10A). (**D**, **E**) Western blot analysis of ZFX protein expression in TNBC cells following transfection with either a miR-218 mimic or miRNA mimic control. (**F**) Relative ZFX mRNA expression following transfection of TNBC cells with either a miR-218 mimic or miRNA mimic control. (**G**, **H**) Luciferase reporter assays demonstrating that overexpression of miR-218 suppresses luciferase activity in MDA-MB-231 and MDA-MB-468 cells transfected with ZFX-Wt. *P < 0.05 compared to controls.

We performed bioinformatics analysis using TargetScan, miRPathDB and starBase 3.0 to identify potential targets of miR-218. A total of eight genes (ARPP19, CCDC6, ZFX, MBNL1, NACC1, PPP1CC, PPP2R2A, and SHOC2) were identified. We reviewed published literature regarding the functions of these eight genes and found that ZFX [28, 29] and NACC1 [30, 31] have been shown to promote cancer cell proliferation and invasion. We transfected TNBC cells with a miR-218 mimic and performed qRT-PCR to determine whether miR-218 regulated the expression of ZFX or NACC1. The expression of NACC1 was not altered following miR-218 overexpression in either MDA-MB-231 or MDA-MB-468 cells (Supplementary Figure 1). Previous studies demonstrated that ZFX plays an essential role in cancer initiation and progression [32]. Therefore, we selected it for further analysis. We identified a putative miR-218 binding site in the 3’-UTR of ZFX through bioinformatics analysis (Figure 6A). We then evaluated the ZFX expression in TNBC compared to adjacent normal tissue using qRT-PCR. ZFX was upregulated in TNBC tissue and cell lines (Figure 6B and 6C, P < 0.05). We transfected TNBC cells with a miR-218 mimic and analyzed endogenous ZFX expression by western blotting and qRT-PCR. These results confirmed that ZFX expression was downregulated by miR-218 in TNBC. Overexpression of miR-218 resulted in downregulation of ZFX expression (Figure 6D–6F, P < 0.05).

We confirmed an interaction between ZFX and miR-218 using luciferase activity assays. Luciferase reporter constructs were generated that expressed either wild-type ZFX (ZFX-Wt) or a mutant version of ZFX in which the miR-218 binding sites were mutated (ZFX-Mut). Overexpression of miR-218 resulted in reduced luciferase activity in TNBC cells transfected with ZFX-Wt but not in cells transfected with ZFX-Mut (Figure 6G and 6H, P < 0.05). These data suggested that miR-218 could directly target ZFX in TNBC.

### MiR-218 inhibits TNBC cell proliferation, migration, and invasion by targeting ZFX

**Figure 7:**
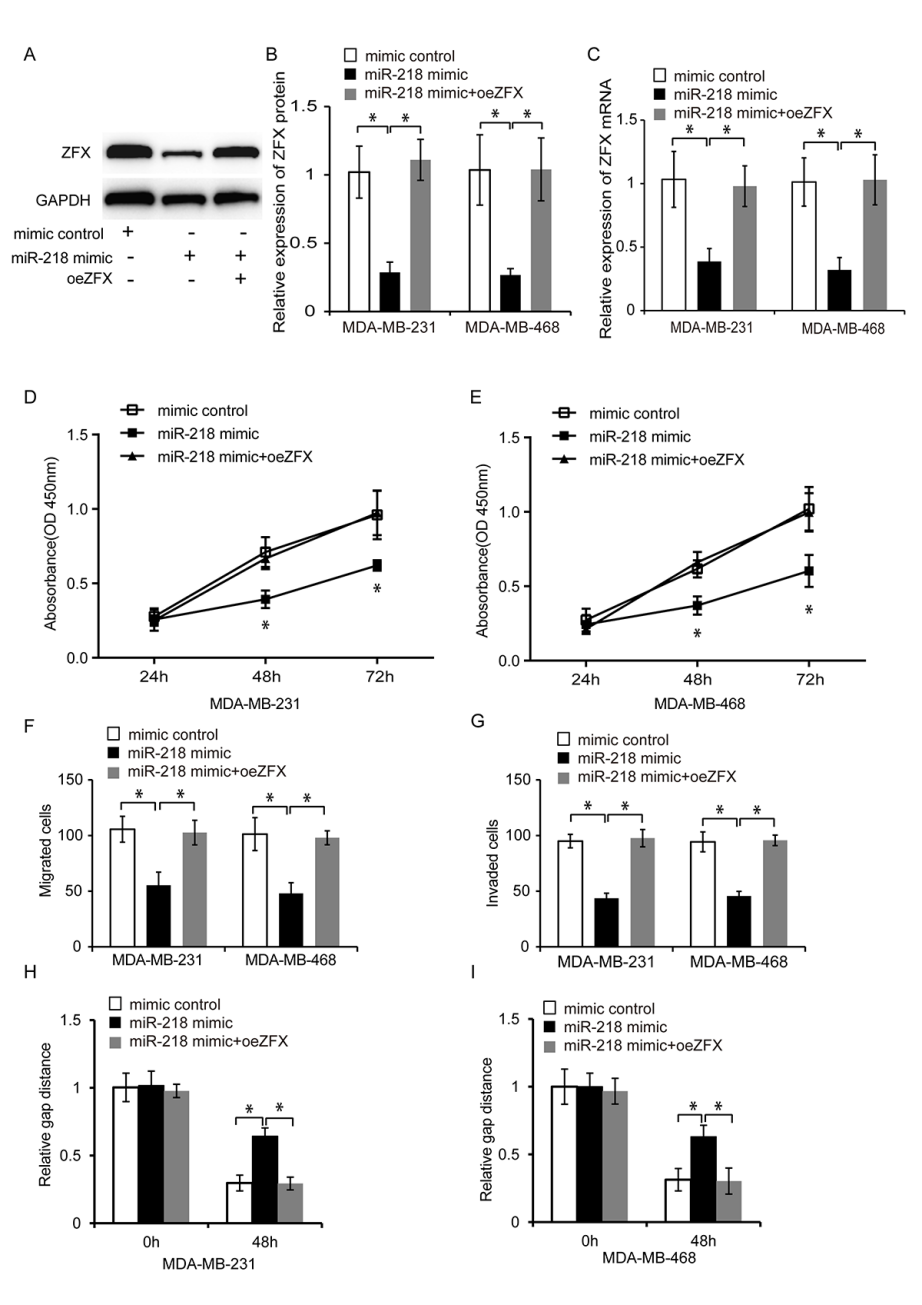
**MiR-218 inhibits TNBC cell proliferation, migration, and invasion by inhibiting ZFX.** (**A**) Representative western blot analysis of TNBC cells following transfection with a miRNA mimic control, miR-218 mimic, or miR-218 mimic plus oeZFX. (**B**) Western blot analysis of the relative ZFX protein expression in TNBC cells following transfection with a miRNA mimic control, miR-218 mimic, or miR-218 mimic plus oeZFX. (**C**) Relative expression of ZFX mRNA in TNBC cells following transfection with a miRNA mimic control, miR-218 mimic, or miR-218 mimic plus oeZFX. (**D**, **E**) Analysis of MDA-MB-231 and MDA-MB-468 cell proliferation following transfection with either a miRNA mimic control, miR-218 mimic, or miR-218 mimic plus oeZFX using CCK8 assays. (**F**, **G**) Analysis of the migration and invasion capacities of MDA-MB-231 and MDA-MB-468 cells transfected with a miRNA mimic control, miR-218 mimic, or miR-218 mimic plus oeZFX using transwell assays. (**H**, **I**) Analysis of the migration capacities of MDA-MB-231 and MDA-MB-468 cells transfected with a miRNA mimic control, miR-218 mimic, or miR-218 mimic plus oeZFX. *P < 0.05 compared to controls.

We next examined whether miR-218 negatively regulated ZFX to suppress TNBC cell proliferation, migration, and invasion. ZFX was overexpressed in TNBC cells transfected with a miR-218 mimic. The transfection efficiency in the control, miR-218 mimic, and miR-218 mimic plus pcDNA-ZFX (oeZFX) groups was evaluated by western blotting and qRT-PCR (Figure 7A–7C, P < 0.05). Cell proliferation, migration, and invasion were then analyzed using CCK8 assays, wound healing assays, and transwell assays, respectively. Overexpression of ZFX abolished the suppressive effects of miR-218 on TNBC cell proliferation, migration, and invasion, suggesting that miR-218 negatively regulates ZFX to inhibit TNBC progression (Figure 7D–7I, P < 0.05).

### CCAT1 promotes tumor growth in a xenograft mouse model

**Figure 8:**
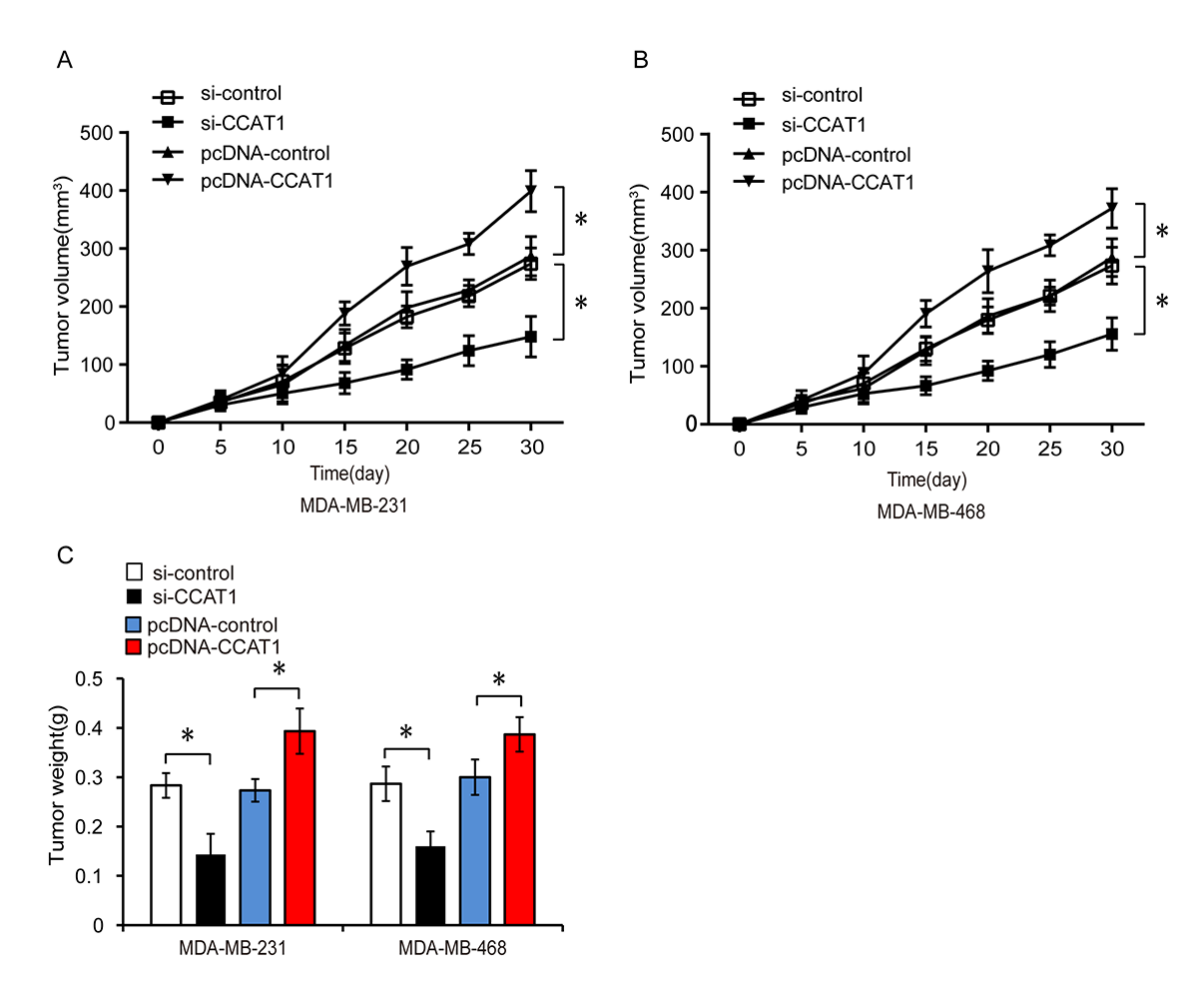
**CCAT1 promotes tumor growth in a xenograft mouse model of TNBC.** (**A**, **B**) Xenograft tumor volume was measured every 5 days among the different treatment groups consisting of MDA-MB-231 or MDA-MB-468 cells transfected with si-control, si-CCAT1, pcDNA-control, or pcDNA-CCAT1. (**C**) Comparison of tumor weight between groups. *P < 0.05 compared to controls.

Because CCAT1 promoted TNBC cell proliferation, migration, and invasion *in vitro*, we investigated whether it could promote tumor growth *in vivo*. We subcutaneously injected nude mice with MDA-MB-231 and MDA-MB-468 cells that were stably transfected with a control siRNA (si-control), siRNA targeting CCAT1 (si-CCAT1), empty vector control (pcDNA-control), or vector expressing CCAT1 (pcDNA-CCAT1) and monitored the growth of the xenograft tumors (Figure 8A–8C, P < 0.05). Tumor weight and volume were lower in the si-CCAT1 compared to the si-control group. Tumor size and weight were higher in the pcDNA-CCAT1 compared to the pcDNA-control group. These results suggested that CCAT1 promotes TNBC progression *in vivo*.

## DISCUSSION

Dysregulation of lncRNAs, including DSCAM-AS1, SNHG15, and HOXA1-AS, has been implicated in breast cancer development and progression, and is correlated with prognosis [15, 33, 34]. However, the roles of most lncRNAs in TNBC progression have not been elucidated. Here, we investigated the potential role of the lncRNA CCAT1 in TNBC. Interestingly, we found that CCAT1 promotes TNBC cell proliferation, migration, and invasion, suggesting it could be a potential therapeutic target for the treatment of this aggressive subtype of breast cancer.

Previous studies suggested that CCAT1 promotes tumor progression in a variety of human cancers. For example, it promotes cell proliferation, migration, and invasion in gastric cancer by targeting Bmi1 and miR-219-1 [24, 25, 35]. Additionally, CCAT1 was shown to promote ovarian cancer progression by regulating miR-1290 [36] and miR-490 [37]. CCAT1 also enhanced proliferation, migration, and invasion in prostate [38], colon [23], and pancreatic cancer [39]. Overexpression of CCAT1 was associated with a poor prognosis among breast cancer patients [26]. We observed upregulation of CCAT1 expression in TNBC cell lines and patient tissue. Increased expression promoted TNBC cell proliferation, migration, and invasion *in vitro*, and tumor progression *in vivo*.

LncRNAs can function as sponges for endogenous miRNAs [40]. For example, CCAT1 was shown to promote tumor progression by functioning as a sponge for miR-490-3p [37], miR-1290 [36], miR-219-1 [25], and miR-181a [41]. We therefore performed a bioinformatics analysis to identify potential miRNA targets of CCAT1 in TNBC. Interestingly, miR-218 was identified as a potential binding partner for CCAT1. Consistent with these results, previous studies showed that CCAT1 promotes proliferation and invasion in several cancers, including gallbladder [42], laryngeal squamous cell carcinoma [43], and retinoblastoma [44], by negatively regulating miR-218-5p. Yang et al. found that miR-218 functions as a tumor suppressor by regulating IL-6/STAT3 signaling and is frequently downregulated in lung cancer [45]. MiR-218 was also found to inhibit epithelial-mesenchymal transition (EMT), migration, and invasion by targeting SFMBT1 and DCUN1D1, and was downregulated in cervical cancer [46]. Finally, miR-218 was shown to inhibit tumor cell invasion and migration by regulating ROBO1 in pancreatic cancer [47].

Several studies have demonstrated that miRNAs play a role in TNBC progression. For example, miR-218-5p/Wnt signaling was found to promote metastasis in TNBC [48]. Additionally, Setijono et al. demonstrated that miR-218 and miR-129 regulate breast cancer progression by targeting lamin proteins [49]. Consistent with earlier studies, we found that miR-218 expression is downregulated in TNBC, and that miR-218 is a downstream target of CCAT1 [48, 49]. CCAT1 knockdown resulted in an increase in miR-218 expression. Overexpression of miR-218 inhibited TNBC cell proliferation, migration, and invasion. Furthermore, knockdown of miR-218 reversed CCAT1 silencing-induced inhibition of TNBC cell proliferation, migration, and invasion. These data suggest that CCAT1 targets miR-218 to promote TNBC progression.

Our findings indicate that miR-218 suppresses TNBC progression by negatively regulating the zinc finger transcription factor ZFX. ZFX has been implicated in various cancers including pancreatic [50], gastric [51], hepatocellular carcinoma [52], malignant glioma [53], and gallbladder cancer [54]. Silencing ZFX suppressed breast cancer cell proliferation [55]. We observed upregulation of ZFX expression in human TNBC cell lines and tissue. Overexpression of ZFX reversed the inhibitory effects of miR-218 on TNBC cells, suggesting that miR-218 inhibits TNBC cell proliferation, migration, and invasion by negatively regulating ZFX.

In summary, our data indicate the CCAT1 promotes TNBC cell proliferation, migration, and invasion by downregulating miR-218 expression. Targeting the CCAT1/miR-218/ZFX signaling pathway may be a potential therapeutic strategy for TNBC treatment.

## METHODS

### Patient tissue specimens

Written informed consent was obtained from all patients prior to the collection of excess tissue specimens. The study was approved by the Ethics Committee of Tianjin Medical University Cancer Institute and Hospital (P.R. China) and was performed in accordance with the Helsinki Declaration. TNBC and adjacent normal breast tissue were collected from 10 patients who were pathologically diagnosed with TNBC (Stage I–IIA) between July 2017 and December 2017 at Tianjin Medical University Cancer Institute and Hospital. None of the patients received chemotherapy, radiotherapy, or hormone therapy prior to surgical resection of the tumors. All tissue specimens were immediately immersed in liquid nitrogen and stored at −80°C.

### Cell lines, culture, and transfection

Human TNBC cell lines (MDA-MB-231, MDA-MB-436, and MDA-MB-468) and a normal breast cell line (MCF-10A) were obtained from ATCC (Manassas, VA, USA) and maintained in Dulbecco’s Modified Eagle’s Medium (DMEM; Gibco, Grand Island, NY, USA) supplemented with 10% fetal bovine serum (FBS; Gibco), 100 U/ml penicillin (Gibco), and 100 U/ml streptomycin (Gibco). The cells were incubated at 37°C in a humidified atmosphere with 5% CO_2_. The pcDNA3.1-CCAT1 and control pcDNA3.1 plasmids were synthesized by GenePharma (Shanghai, China). The siRNAs (si-CCAT1 and si-control) were designed and synthesized by Ribobio Co., Ltd. (Guangzhou, P.R. China). The miR-218 mimic, inhibitor, and corresponding negative controls were also synthesized by Ribobio Co., Ltd. (Guangzhou, P.R. China). Cells were transfected using Lipofectamine 2000 (Invitrogen, Carlsbad, CA, USA) according to the manufacturer’s instructions. The transfection efficiency was evaluated by qRT-PCR.

### CCK8 assays of cell proliferation

MDA-MB-231 and MDA-MB-468 cells were incubated at 37°C for 24, 48, or 72 hours and cell proliferation analyzed using CCK-8 assays according to the manufacturer’s instructions (CCK-8; Sigma-Aldrich, St. Louis, MO, USA).

### *In vitro* migration and invasion assays

For migration assays, breast cancer cell lines (1 × 10^5^ cells) were suspended in serum-free media and seeded into the upper chambers of transwell chambers (Corning, Corning, NY, USA). For invasion assays, breast cancer cell lines were seeded into the upper chambers of transwell inserts that were precoated with Matrigel (BD Biosciences, San Jose, CA, USA). The lower chambers were filled with 500 μl of DMEM containing 10% FBS and the cell incubated at 37 °C for either 12 hours (migration assays) or 24 hours (invasion assays). Following the incubation, the cells in the upper chambers were removed using cotton swabs. The cells at the bottom of the membranes were fixed with 3.7% formaldehyde, stained with 0.5% crystal violet for 20 min, washed with PBS, and counted under a light microscope.

### Wound healing assays

Cell migration was analyzed using wound healing assays. Following transfection, cells were seeded into six well plates (4×10^5^ cells/well) and cultured until they reached approximately 90% confluence. Wounds were generated by scratching cell monolayers using a 200 μl pipette tip. Cells were then cultured in media containing 1% FBS for 48 hours. Images were detected using an inverted microscope (Olympus, Japan) at 100×magnifcation.

### Quantitative RT-PCR

Total RNA was extracted from human tissue specimens and cultured cells using the TRIzol reagent (Invitrogen) according to the manufacturer's instructions. Complementary DNA was then synthesized using the M-MLV reverse transcriptase (Promega, Madison, WI, USA). The relative expression of CCAT1 and miR-218 was evaluated by qRT-PCR using the SYBR Green detection system and a 7500 Real Time PCR System (Applied Biosystems). U6 was used as the normalization control for miR-218 and GAPDH for CCAT1.

### Luciferase reporter assays

MDA-MB-231 and MDA-MB-468 cells were co-transfected with either a wild-type or mutant CCAT1 reporter plasmid, and either a miR-218 mimic or a miRNA mimic control according to the manufacturer’s instructions. Luciferase activity was measured 48 hours after transfection using the Dual-Luciferase^®^ Reporter Assay System (Promega, Madison, WI, USA) according to the manufacturer’s protocol.

### Western blot analysis

Breast cancer cells were lysed in RIPA buffer (Beyotime Institute of Biotechnology, Beijing, China) and total protein extracted. The protein concentrations were estimated using a BCA Protein Assay Kit (Beyotime Institute of Biotechnology, Beijing, China). Equal quantities of protein (30 μg) were separated by 10% SDS-PAGE and then electrotransferred onto PVDF membranes (Millipore, Boston, MA, USA). The membranes were first blocked with 5% nonfat milk in Tris-buffered saline containing 0.1% Tween 20 (TBST) and then incubated at 4°C overnight with a primary antibody against ZFX (Sigma-Aldrich, St. Louis, MO, USA). After washing three times with TBST, the membranes were incubated with horseradish peroxidase-conjugated secondary antibodies (Santa Cruz Biotechnology, Dallas, TX, USA). The bands were visualized using the Enhanced Chemiluminescence Kit (GE Healthcare, Chicago, IL, USA).

### *In vivo* mouse model

Nude mice (4–6 weeks old, female) were maintained under pathogen free conditions. All protocols were approved by the Animal Care Committee of Tianjin Medical University. For the xenograft tumor experiments, 1×10^7^ stable MDA-MB-231 or MDA-MB-468 cells transfected with pcDNA-control, pcDNA-CCAT1, si-control, or si-CCAT1 were subcutaneously injected into mice (n = 3 per group). Xenograft tumors were measured every 5 days. Tumor volume was calculated using the following formula: length × width^2^ × 0.5. Mice were sacrificed after 30 days.

### Statistical analysis

All data are presented as the mean ± standard deviation. Statistical analysis was performed using SPSS 17.0 (SPSS Inc., Chicago, IL, USA) and GraphPad Prism 7.0 (San Diego, CA, USA). Comparisons between groups were performed using Student’s unpaired t-tests. A value of P < 0.05 was considered statistically significant.

## Supplementary Material

Supplementary Figure 1
