# Supplementary material for: CCAT1 promotes triple-negative breast cancer progression by suppressing miR-218/ZFX signaling
Source: Aging (Albany NY). 2019 Jul 16;11(14):4858–75. doi: 10.18632/aging.102080 (PMC6682511; doi:10.18632/aging.102080)
Supplement: Supplementary Figure 1 [file aging-11-102080-s001.pdf]

SUPPLEMENTARY FIGURES

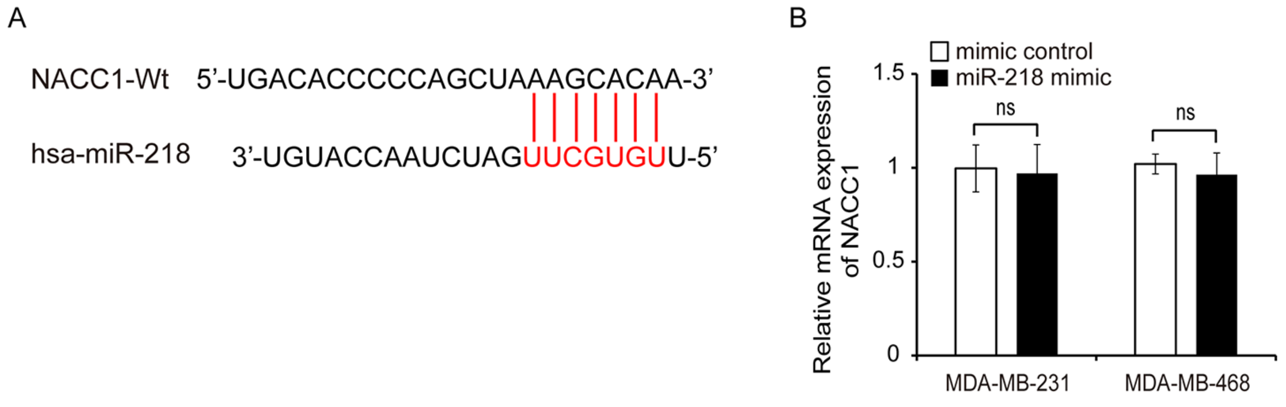

Supplementary Figure 1. MiR-218 does not target NACC1 in TNBC. (**A**) Diagram showing the predicted miR-218 binding site in the NACC1 sequence. (**B**) Relative NACC1 mRNA expression in TNBC cells following transfection with either a miR-218 mimic or miRNA mimic control. ns, not statistically significant.
